# Analysis of the Knockdown Resistance Locus (*kdr*) in *Anopheles stephensi*, *An. arabiensis*, and *Culex pipiens s.l.* for Insight Into the Evolution of Target-site Pyrethroid Resistance in Eastern Ethiopia

**DOI:** 10.4269/ajtmh.20-1357

**Published:** 2022-01-10

**Authors:** Tamar E. Carter, Araya Gebresilassie, Shantoy Hansel, Lambodhar Damodaran, Callum Montgomery, Victoria Bonnell, Karen Lopez, Daniel Janies, Solomon Yared

**Affiliations:** ^1^ Department of Biology, Baylor University, Waco, Texas;; ^2^ Department of Zoological Sciences, Addis Ababa University, Addis Ababa, Ethiopia;; ^3^ Department of Bioinformatics and Genomics, University of North Carolina at Charlotte, Charlotte, North Carolina;; ^4^ Institute of Bioinformatics, University of Georgia, Athens, Georgia;; ^5^ Department of Molecular Biology and Biochemistry, Pennsylvania State University, State College, Pennsylvania;; ^6^ Department of Biology, Jigjiga University, Jigjiga, Ethiopia

## Abstract

The malaria vector, *Anopheles stephensi*, which is typically restricted to South Asia and the Middle East, was recently detected in the Horn of Africa. Addressing the spread of this vector could involve integrated vector control that considers the status of insecticide resistance of multiple vector species in the region. Previous reports indicate that the knockdown resistance mutations (*kdr*) in the voltage-gated sodium channel (*vgsc*) are absent in both pyrethroid-resistant and pyrethroid-sensitive *An. stephensi* in eastern Ethiopia; however, similar information about other vector species in the same areas is limited. In this study, *kdr* and the neighboring intron were analyzed in *An. stephensi*, *An. arabiensis*, and *Culex pipiens *s.l. collected between 2016 and 2017 to determine the evolutionary history of *kdr* in eastern Ethiopia. A sequence analysis revealed that all of *Cx. pipiens *s.l. (*N* = 42) and 71.6% of the *An. arabiensis* (*N* = 67) carried *kdr* L1014F, which is known to confer target-site pyrethroid resistance. Intronic variation was only observed in *An. stephensi* (six segregating sites, three haplotypes), which was previously shown to have no *kdr* mutations. In addition, no evidence of non-neutral evolutionary processes was detected at the *An. stephensi kdr* intron, thereby further supporting the target-site mechanism not being a major resistance mechanism in this *An. stephensi* population. Overall, these results show key differences in the evolution of target-site pyrethroid/dichlorodiphenyltrichloroethane resistance mutations in populations of vector species from the same region. Variations in insecticide resistance mechanism profiles between eastern Ethiopian mosquito vectors may lead to different responses to insecticides used in integrated vector control.

## INTRODUCTION

Vector-borne diseases are a major public health concern; of these, malaria remains a leading threat, with 229 million cases reported in 2019.^1^ In Ethiopia, where both *Plasmodium vivax* and *P. falciparum* are prevalent and multiple *Anopheles* vector populations are present, 2.6 million malaria cases were reported in 2019.^1^ Malaria control in Ethiopia and the rest of Africa is now challenged with the recent discovery of *An. stephensi*, a malaria vector that is typically restricted to South Asia and the Middle East, in the Horn of Africa that recently demonstrated local transmission of *Plasmodium.*^2^^–^^5^ Among several approaches to mitigating the spread of *An. stephensi* is integrated vector control that targets multiple vectors. Integrated vector control has the benefits of reducing costs and minimizing adverse outcomes of single-target vector control for nontarget species populations.^6^

Integrated vector control strategies based on insecticides should account for insecticide resistance status of the different vectors. In Ethiopia, insecticides like pyrethroids have been deployed through indoor residual spraying and long-lasting insecticidal nets (LLIN). Exacerbated by the use of insecticides in agricultural industries, widespread insecticide resistance has evolved and has been reported across multiple vector species.^7^ In Culicidae, the main mechanisms of resistance to pyrethroids include target-site and metabolic-based resistance.^8^ Pyrethroid-based target-site resistance is caused by mutations in the voltage-gated sodium channel that lead to an altered neurological response to insecticides in mosquitoes (i.e., knockdown resistance [*kdr*]).^9^ Knockdown resistance is broadly studied and is widely reported across species of Culicidae, including *Anopheles* spp.^8^ and *Culex pipiens *s.l.^10^ Regarding *Anopheles*, *kdr* involves the substitution of leucine (TTA) with phenylalanine (TTT) or serine (TCA) in the voltage-gated sodium channel protein, commonly known as *kdr* mutations L1014F and L1014S.^11^ Similar mutations that confer resistance to pyrethroids (also known as L1014F and L1014S) are observed in the *vgsc* of *Culex* mosquitoes.

To achieve metabolic resistance, the insecticide is degraded, sequestered, or exported out of the cell before it can bind to its target.^8^ Metabolic resistance has not been linked to a single trackable genetic variant in most species. However, previous functional studies have found that overexpression of detoxification enzymes such as cytochrome P450s leads to metabolic resistance.^8^^,^^12^

In Ethiopia, pyrethroid resistance and dichlorodiphenyltrichloroethane (DDT) resistance have been reported for the primary malaria vector *An. arabiensis* in much of the northern and western portions of the country.^13^^–^^16^ Regarding *An. arabiensis*, both target-site and metabolic resistance have a role in pyrethroid resistance and DDT resistance. In eastern Ethiopia, a recent investigation revealed that *An. stephensi* was resistant to pyrethroids and DDT (consistent with findings outside of Ethiopia^17^); however, the L1014F and L1014S mutations were absent.^18^ *An. arabiensis* insecticide resistance in eastern Ethiopia has not been well-characterized. Furthermore, the status of insecticide resistance in *Cx. pipiens *s.l. (most likely *Cx. p. quinquefasciatus*) is unknown throughout most of the country.

Knowing the status of resistance to pyrethroids across vector species in a region can provide insight into the effectiveness of particular insecticides used to target multiple species. Genetic analyses of putative insecticide resistance loci across local vector populations can provide information about the range of mechanisms of insecticide resistance in a region. Although *kdr* L1014F and L1014S mutation frequencies provide preliminary evidence of target-site resistance to pyrethroids, an analysis of the variations in neighboring intronic regions provides information about the long-term impact of pyrethroids on the evolution of the mosquito populations. Tests of neutrality^19^ can be used to evaluate the genetic diversity of the *kdr* locus, including the intronic region, to determine if the patterns differ from expectations under neutral evolution. It is expected that if the *kdr* locus undergoes selection because of pressure from the pyrethroids, then we would hypothesize that a selective sweep would lead to decreased nucleotide diversity of linked alleles via hitchhiking.^20^^,^^21^ Therefore, these analyses are helpful to clarifying the mechanisms of resistance, clarifying the current status of pyrethroid resistance, and predicting the risk of resistance emerging locally. We examined the nucleotide diversity surrounding the *kdr* locus to test the hypothesis of selective sweeps in *An. stephensi*,* An. arabiensis*, and *Culex pipiens *s.l. collected in eastern Ethiopia.

## METHODS

This study involved sequencing of a portion of the *vgsc* gene that contains loci that, when mutated, confer resistance to pyrethroids. For *An. stephensi*, data were from sequences generated during a previous study^18^ and the present study. *An. arabiensis* and *Culex* sequence data were also generated during this study.

### Sample collection and species identification.

**Figure 1.**
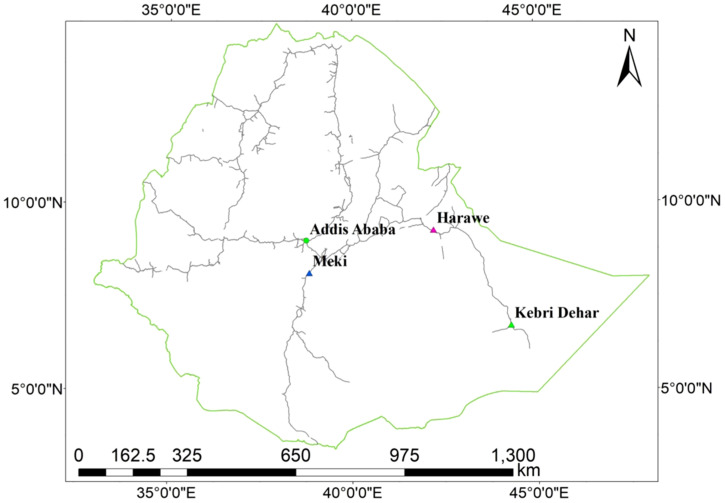
Collection sites. This figure appears in color at www.ajtmh.org.

*An. stephensi* was collected from Kebri Dehar during the 2016 survey that resulted in the first identification of the species in Ethiopia.^3^ Mosquitoes were collected as larvae and laboratory-reared for testing for resistance to insecticides as previously detailed.^18^ *An. arabiensis* and *Culex* specimens collected in eastern Ethiopia in 2017 were included in this study. *An. arabiensis* species identification was based on morphological keys and molecular analysis of internal transcribed spacer 2 (*ITS2*) and cytochrome oxidase I (*COI*) loci, as reported previously.^22^ *An. arabiensis* were collected using CDC light traps (John W. Hock, Gainesville, FL) over four different collection times at two sites, Meki (east-central Ethiopia) and Harewe (east) in 2017. Harewe and Meki are approximately 350 km and 600 km northwest of Kebri Dehar, respectively (Figure 1).

*Culex* specimens were collected using CDC light traps in Kebri Dehar in 2017. The morphological key and sequencing of the *ITS2* locus were used for *Culex* identification using a previously published polymerase chain reaction (PCR) protocol.^3^ All amplicons were cleaned using Exosap and sequenced using Sanger technology with ABI BigDyeTM Terminator version 3.1 chemistry (Thermo Fisher, Santa Clara, CA) according to recommendations of the manufacturer and run on a 3130 Genetic Analyzer (Thermo Fisher). Sequences were cleaned and analyzed using CodonCode Aligner Program version 6.0.2 (CodonCode Corporation, Centerville, MA). ITS2 sequences from *Culex* specimen were submitted as queries to the National Center for Biotechnology Information’s Basic Local Alignment Search Tool for species identification.^23^

### Amplification and sequencing of kdr loci.

When species identification or species complex identification was complete, samples were processed. For the *kdr* mutation analysis, PCR was used to amplify the region of the *vgsc* gene that housed the homologous *kdr* 1014 and a neighboring downstream intron in all specimens (reference sequences used for *An. stephensi*, *An. arabiensis*, and *Culex pipiens *s.l. were JF304952, GU248311, and BN001092, respectively). One leg from each mosquito specimen or extracted DNA was used as an individual template for PCR. Each species required a different PCR protocol. DNA extraction were performed using DNeasy Qiagen kit (Qiagen, Valencia, CA). All PCRs were performed with 25 µL with 12.5 µL 2X Promega Hot Start Master Mix (Promega Corporation, Madison, WI) and the primer conditions listed in Table 1. *An. stephensi kdr* amplification was completed according to Singh et al.,^24^ with modifications detailed by Yared et al.^18^ Temperature cycling was as follows: 95°C for 5 minutes, followed by 35 cycles of 95°C for 30 seconds, 50°C for 30 seconds, 72°C for 45 seconds, and a final extension of 72°C for 7 minutes. Amplifications of the *kdr* fragment from *An. arabiensis* were completed according to the methods of Verhaeghen et al.^25^ Temperature cycling was as follows: 95°C for 1 minute, followed by 30 cycles of 95°C for 30 seconds, 52°C for 30 seconds, 72°C for 1 minute, and a final extension of 72°C for 10 minutes. Amplifications of the *kdr* fragment from *Culex pipiens* s.l. were completed according to the methods of Chen et al.^26^ Temperature cycling was as follows: 94°C for 5 minutes, followed by 30 cycles of 94°C for 40 seconds, 58°C for 30 seconds, 72°C for 40 seconds, and a final extension of 72°C for 8 minutes.

**Table 1:** List of primers and conditions used for polymerase chain reaction amplification of portions of the voltage-gated sodium channel gene

Assay	Primer	Sequence	Annealing temperature (°C)	Final primer concentration (µM)
*An. stephensi*	*Kdr*F	GGACCAYGATTTGCCAAGAT	50	1.25
	VGS_1R	CGAAATTGGACAAAAGCAAGG	50	1.25
*An. arabiensis*	Agd1	ATAGATTCCCCGACCATG	52	1.25
	Agd2	AGACAAGGATGATGAACC	52	1.25
*Culex*	Cpp1	CCTGCCACGGTGGAACTTC	58	1
	Cpp2	GGACAAAAGCAAGGCTAAGAA	58	1

All amplicons were cleaned using Exosap and sequenced using Sanger technology with ABI BigDyeTM Terminator version 3.1 chemistry (Thermo Fisher) according to the manufacturer’s recommendations and run on a 3130 Genetic Analyzer (Thermo Fisher).

### Sequence analysis.

Sequences were submitted as queries to the National Center for Biotechnology Information’s Basic Local Alignment Search Tool to confirm that the correct loci were amplified. Sequences were then aligned in CodonCode (CodonCode Corp., Dedham, MA) according to species or species complex to identify *kdr* L1014F or L1014S mutations based on reference sequence details from previous reports.^18^^,^^24^^,^^25^ Heterozygous genotypes at *kdr* were determined based on the number of peaks observed in the chromatogram, with each peak indicating different alleles. The *kdr* allele and genotype frequencies were then calculated and compared across species.

We determined the level of diversity in the neighboring intron downstream of the *kdr* 1014 in *Culex* spp., *An. arabiensis*, and* An. stephensi* for additional evidence of selection on that locus. In addition to the sequences generated in this study, we included sequences from resistant and nonresistant *An. stephensi* analyzed during a previous study of insecticide resistance in *An. stephensi.*^18^ We calculated the number of segregating sites, nucleotide diversity, estimated number of haplotypes, and haplotype diversity using the program DNAsp version 5.^27^ Haplotypes were reconstructed using Phase 2.1,^28^ HAPAR, and fastPHASE^29^ algorithms in DNAsp. The neighboring downstream intron was also tested for neutrality using Tajima’s D,^19^ Fu’s F,^30^ and Fu and Li’s D* and F* tests.^31^

## RESULTS

Before insecticide resistance genotyping, all *Culex* ITS2 sequences were analyzed to identify species. All sequences were identical and had equivalent high matching scores for two members of the *Cx. pipiens* complex: *Cx. p. quinquefasciatus* and *Cx. p. pipiens*. Because we could not identify the species of these mosquitos, we refer to these specimens by the broader taxonomic classification, *Cx. pipiens s.l.* (i.e., *Cx. pipiens* complex), in this study. *An. arabiensis* species identification was detailed in a previous study.^22^ Totals of 10, 33, and 24 *An. arabiensis* were collected in Harewe in November 2016, Harewe in July/August 2017, and Meki in July 2017, respectively.

### The *kdr* analysis.

**Figure 2.**
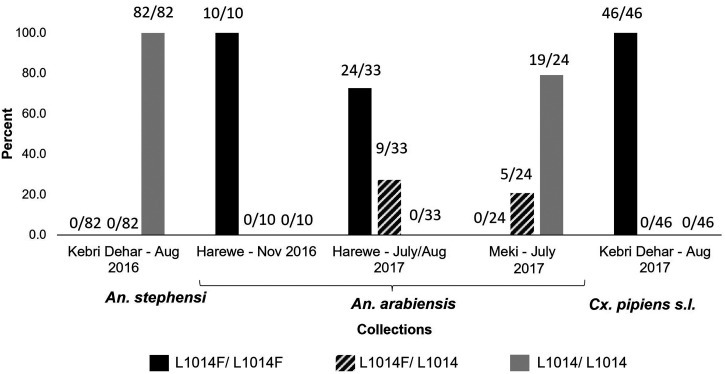
Frequency of *kdr* 1014 genotypes in *An. stephensi*,* Culex pipiens *s.l., and *An. arabiensis* collections.

The *kdr* fragments were sequenced for *An. stephensi*, *Cx. pipiens *s.l., and *An. arabiensis.* The sequencing resulted in 184, 452, and 290 basepair fragments for *An. stephensi*, *Cx. pipiens *s.l., and *An. arabiensis*, respectively. The percent of each *kdr* genotype observed by species is shown in Figure 2. A total of 131 *An. stephensi* were analyzed, including 80 newly reported sequences. None of the *An. stephensi* analyzed during this study had a mutation at *kdr* 1014. All 42 *Cx. pipiens *s.l. specimens collected at the same site carried *kdr* L1014F mutations as homozygous. Of the 67 *An. arabiensis*, 71.6% carried the *kdr* L1014F mutation (heterozygous and homozygous). The allele frequency of L1014F mutation varied across *An. arabiensis* collections, in which the highest frequency was observed in Harewe in November 2016 (100%). The L1014F allele frequencies for the Harewe July/August 2017 and Meki July 2017 collections were 86.4% and 10%, respectively. No L1014S mutations were detected in *Cx. pipiens* s.l. or *An. arabiensis*.

**Figure 3.**
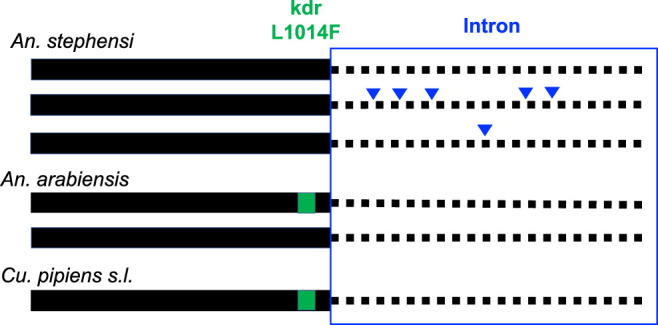
Summary of *kdr* haplotypes across three Culicidae species in eastern Ethiopia. Solid lines depict the exon housing of the *kdr* locus and dotted lines depict the downstream intron. Green square indicates the presence of *kdr* L1014F. Triangles denote single nucleotide polymorphisms (SNPs) found in the intron relative to the most prevalent intron haplotype. This figure appears in color at www.ajtmh.org.

Some of the neighboring downstream introns for each species were analyzed to evaluate the level of diversity (Figure 3). The intron analysis revealed no polymorphisms for either *Cx. pipiens* or *An. arabiensis* (for both L1014F and L1014 wild type specimens). Of the 131 *An. stephensi* specimens from Kebri Dehar examined for *kdr* mutations, six segregating sites were detected and three haplotypes were predicted. Genetic diversity estimates are reported in Table 2.

**Table 2:** Genetic diversity estimates for *kdr* neighboring downstream introns in the *vgsc* for *An. stephensi*, *An. arabiensis*, and *Cx. pipiens *s.l.

Species	n	S	k	Pi	h	Hd
*An. stephensi*	262	6	0.996	0.00545	3	0.225
*An. arabiensis*	134	0	0	0	1	0
*Cx. pipiens *s.l.	84	0	0	0	1	0

n = number of genes (two per individual); S = number of polymorphic (i.e., segregating) sites; K = average number of pairwise nucleotide differences; Pi = nucleotide diversity; h = number of haplotypes; Hd = haplotype diversity.

To further evaluate the potential functional significance of the *kdr* locus in *An. stephensi* based on evidence of positive selection, we performed neutrality tests at the *An. stephensi kdr* intron. No evidence of non-neutral processes was detected in *An. stephensi* for the *kdr* locus (Table 3). The absence of variations in *An. arabiensis* and *Cx. pipiens* s.l*. kdr* introns precluded neutrality tests.

**Table 3:** Neutrality tests for downstream *kdr* introns for *An. stephensi*

Test	Estimate
n	258
Tajima’s D	0.03839
Fu’s F	3.556
Fu and Li’s D	1.04354
Fu and Li’s F	0.82943

All *P* > 0.10.

## DISCUSSION

Our results revealed variations at the *kdr* locus across different vector species found in eastern Ethiopia that suggest that the role of the target-site mechanism in pyrethroid/DDT resistance varies across species. Notably, the *kdr* L1014F mutation was not observed consistently across the species included in this study. Unlike the *An. stephensi*, which carried no L1014F mutations, both *Cx. pipiens *s.l. and *An. arabiensis* carried L1014F. Based on these findings, it is likely that *Cx. pipiens *s.l. and *An. arabiensis* do not share the same resistance mechanism profile as *An. stephensi* (i.e., *kdr* target-site resistance is not relevant in all species from a single region). We also observed differences in the nucleotide diversity of the neighboring intronic region of the three species. Although *An. stephensi* exhibited multiple segregating sites and resultant haplotypes, only a single intronic haplotype is observed for *An. arabiensis* and *Cx. pipiens *s.l. These data may point to distinct differences in biological and environmental factors that shape each species/population. From a species standpoint, behaviors shaped by both their biology and environment, like feeding and resting preferences, may impact the degree of exposure to insecticides.^32^ Given the variations in resting behavior among the mosquito species represented here (e.g., *An. arabiensis* is more exophilic^33^^,^^34^ and *An. stephensi* is more endophilic^35^), variations in exposure to insecticides may occur and may impact the emergence of *kdr* mutations. During our study, we expected that endophilic mosquitoes would have greater exposure to insecticides; therefore, *An. stephensi* would have stronger selective pressure on the *kdr* locus, leading to less variation. However, the opposite was observed during our study, which causes us to question the feeding and biting behaviors of eastern Ethiopian *An. stephensi*.^32^ It is unclear if the level of insecticide used in the areas surveyed during this study was enough to impact the biting behavior of *An. stephensi*. More information about eastern Ethiopian *An. stephensi* biting and resting behaviors can elucidate behavioral adaptations to insecticides in *An. stephensi*.

In addition to species-level differences, the different patterns of *kdr* variations may be explained by multiple evolutionary processes acting on each population sampled. The data may reflect different levels of selective pressure occurring at each location, such that the populations that were undergoing selective pressure from insecticides associated with malaria control or agricultural activities exhibited *kdr* mutations and no intronic variation. We know that DDT-based and pyrethroid-based insecticides and pesticides have a long history of use in the country,^15^^,^^36^^,^^37^ although the data about their use at the local level in eastern Ethiopia are quite limited. The variations may also reflect previous demographic events, like recent decreases in population size or population introductions resulting in a bottleneck and a decrease in intronic variation. We can best evaluate these possibilities in the context of variations in other regions of the genomes in these mosquitoes. Cytochrome oxidase subunit I (*COI*) has been previously analyzed in *An. arabiensis* and *An. stephensi.*^3^^,^^22^ Although multiple *COI* haplotypes were observed for each *An. arabiensis* collection,^22^ only a single *COI* haplotype was identified in *An. stephensi*.^3^ The higher level of diversity in *COI* in *An. arabiensis* relative to the *kdr* intronic region supports that selective pressure rather than a population bottleneck has shaped the variation at the *kdr*. The opposite pattern observed in *An. stephensi* provides greater support for the absence of selection on that locus in that species. The degree of variation at *kdr* in *An. stephensi* may also reflect the likely notion of this species being a recent introduction to that region;^38^ therefore, it would not have had the same number of years of exposure to the local pressure that would cause evolved target-site resistance in the local vector populations. No *COI* data were available for the *Cx. pipiens* s.l. in this study, and both population bottleneck and/or selection on the *kdr* locus remain plausible explanations for the lack of variation. The multiple collections that comprised our *An. arabiensis* sample set provide preliminary insight into the basis for population *kdr* variations within a species. We observed a range of *kdr* allele frequencies across the *An. arabiensis* sample collections. The collections differ by location and/or date of collection, suggesting that geography or timing could have a role in the variations in *kdr* L1014F frequencies observed. Insecticide resistance phenotypes and mutations have been shown to vary within a single population,^39^^–^^41^ which may correspond to the seasonal use of insecticides for vector control or agricultural activities. Additional surveillance in a larger sample size is needed to verify the importance of geographic and temporal factors shaping the frequency of the mutation. Another notable observation was the shared intron haplotype between the *An. arabiensis* that carried the L1014F mutation to those that did not. The mosquitoes that carried the once advantageous allele may experience fitness costs in the absence of the selective pressure, which would result in a rebound of the wild-type allele at that locus. These findings highlight the value of investigating the *kdr* intronic variation for evidence of fluctuating selective pressures and the potential for the emergence of insecticide resistance in the future.

Our findings have important implications for the molecular surveillance of target-site pyrethroid resistance mechanisms. An analysis of the *kdr* intronic variation provides important evolutionary historical context to the observed high or low frequency of the *kdr* mutations that have programmatic implications. In this study, whether *kdr* mutations were observed at a high frequency (e.g., Harewe in 2016 and 2017) or low frequency (e.g., Meki in July 2017), low intronic variation may be a signal of past selective pressure and the potential for target-site resistance to emerge rapidly. In the future, molecular surveillance should include an analysis of *kdr* intronic variation to better predict the responses to insecticides by the various vector species present. In addition, comparing *kdr* frequencies across species sheds light on how the introduction of a new vector species population could lead to differing resistance mechanism profiles across the species targeted for integrated vector control. In this case, the absence of *kdr* in the *An. stephensi* population (likely influenced by the variable exposure to insecticides on its path to introduction into eastern Ethiopia) suggests that standing resistance because of metabolic mechanisms would not be compounded by the presence of target-site resistance in *An. stephensi*, the opposite scenario whereby a new vector introduces resistant mutations could be possible in other settings. Therefore, vector control strategies must account for the unique and dynamic population insecticide exposure histories that invasive vectors have compared with native species in the context of local interventions targeting multiple species. An analysis of *kdr* genetic variations will provide critical information that will help achieve this goal.

Several limitations to these studies should be considered. The *An. stephensi* was collected as larvae and pupae and the *An. arabiensis* and *Cx. pipiens *s.l. were collected as wild-caught adults. This method of collection may pose a concern that the immature specimen set would not reflect the natural diversity of the wild-caught adult population. Concerns with clonality, however, are lowered when considering the level of diversity observed at the *An. stephensi kdr* locus and at the ace-1R locus (three haplotypes detected; data not shown). In addition, although *An. stephensi* phenotypic resistance has been reported for eastern Ethiopia, phenotypic data regarding *An. arabiensis* and its association with *kdr* has only been studied for portions of the country outside of eastern Ethiopia. Furthermore, the association of *kdr* mutations and phenotypic resistance in *Cx. pipiens *s.l. observed in other parts of the world have not been confirmed in Ethiopia. Follow-up studies would benefit from additional bioassay tests for *An. arabiensis* and *Cx. pipiens* in eastern Ethiopia in conjunction with the molecular analysis of *kdr*. Finally, because of the geographic variation in *kdr* mutation frequencies observed in *An. arabiensis*, future studies should examine the frequency of *kdr* mutations of these vectors in other regions in Ethiopia to confirm the status of target-site pyrethroid/DDT resistance.

In conclusion, the different patterns of diversity at the *kdr* loci across species support the notion that Culicidae in eastern Ethiopia likely have different profiles of pyrethroid/DDT resistance mechanisms. Both *An. arabiensis* and *Cx. pipiens* sample sets revealed notable L1014F allele frequencies that confer target-site resistance and the absence of intron variations that may be caused by positive selective pressure on that locus in those species. Additional investigations are needed to confirm other resistance mechanisms (metabolic, cuticle, or another undiscovered mechanism) and the genetic basis of pyrethroid resistance in *An. stephensi*. Coordinated studies of agricultural and vector control practices with molecular surveillance would also enhance our ability to evaluate the cause of *kdr* variation observed here. These findings emphasize the need for careful consideration of molecular approaches used to evaluate the insecticide resistance status across multiple species and will inform the development and future implementation of novel integrated vector control strategies.
